# Klotho protein: a new insight into the pathogenesis of essential hypertension

**DOI:** 10.1186/s40885-024-00294-5

**Published:** 2024-12-01

**Authors:** Richa Awasthi, Priyanka Thapa Manger, Rajesh Kumar Khare, Roshan Alam

**Affiliations:** 1 Department of Biochemistry, Integral Institute of Medical Sciences and Research, Integral University, Lucknow, India; 2 Department of Medicine, Integral Institute of Medical Sciences and Research, Integral University, Lucknow, India

**Keywords:** Klotho protein, Essential hypertension, Wingless-related integration site pathway, Fibroblast growth factor-23

## Abstract

**Background:**

Essential hypertension is a most prevalent global health concern. Despite extensive research, the exact mechanisms contributing to essential hypertension remain unclear. Several factors contribute to the pathogenesis of essential hypertension. Klotho, a membrane-bound and soluble protein, has been found to modulate hypertension through physiological processes like vascular function and sodium balance. This study aimed to determine the association of klotho protein with essential hypertension.

**Methods:**

The study included 164 hypertensive cases and 164 normotensive controls, after imposing certain inclusion and exclusion criteria with written consent from all subjects. Subject’s details were obtained using structured proforma to account for potential confounding variables. To estimate klotho protein activity using sandwich enzyme-linked immunosorbent assay, 2 mL blood was collected in a plain vial. All data were tested at a 5% significance level.

**Results:**

The analysis revealed a significant decrease in klotho protein levels in cases compared to controls (1.52 ± 0.87 vs. 2.45 ± 0.90, *P* < 0.001), suggesting an inverse relationship of klotho protein with risk of essential hypertension. All indices in the structural equation model have suggested that the final model fitted the data reasonably (chi-square to df ratio, 1.153; goodness of fit index, 0.990; adjusted goodness of fit index, 0.945; normed fit index, 0.936; standardized root mean square residual, 0.953; root mean square error of approximation, 0.031). Also, klotho was negatively associated with blood pressure. The area under the receiver operating characteristic curve for klotho and blood pressure was 0.765 (95% confidence interval, 0.716–0.815; *P* < 0.001).

**Conclusions:**

Klotho levels were significantly reduced in essential hypertension cases compared to controls, Also, klotho had a negative direct association with essential hypertension indicating a potential role for klotho as a prognostic and predictive marker for essential hypertension. This suggests that klotho may have a role in the pathogenesis of essential hypertension. Understanding klotho’s role in essential hypertension may lead to the development of novel therapeutic strategies for this disease.

**Graphical Abstract:**

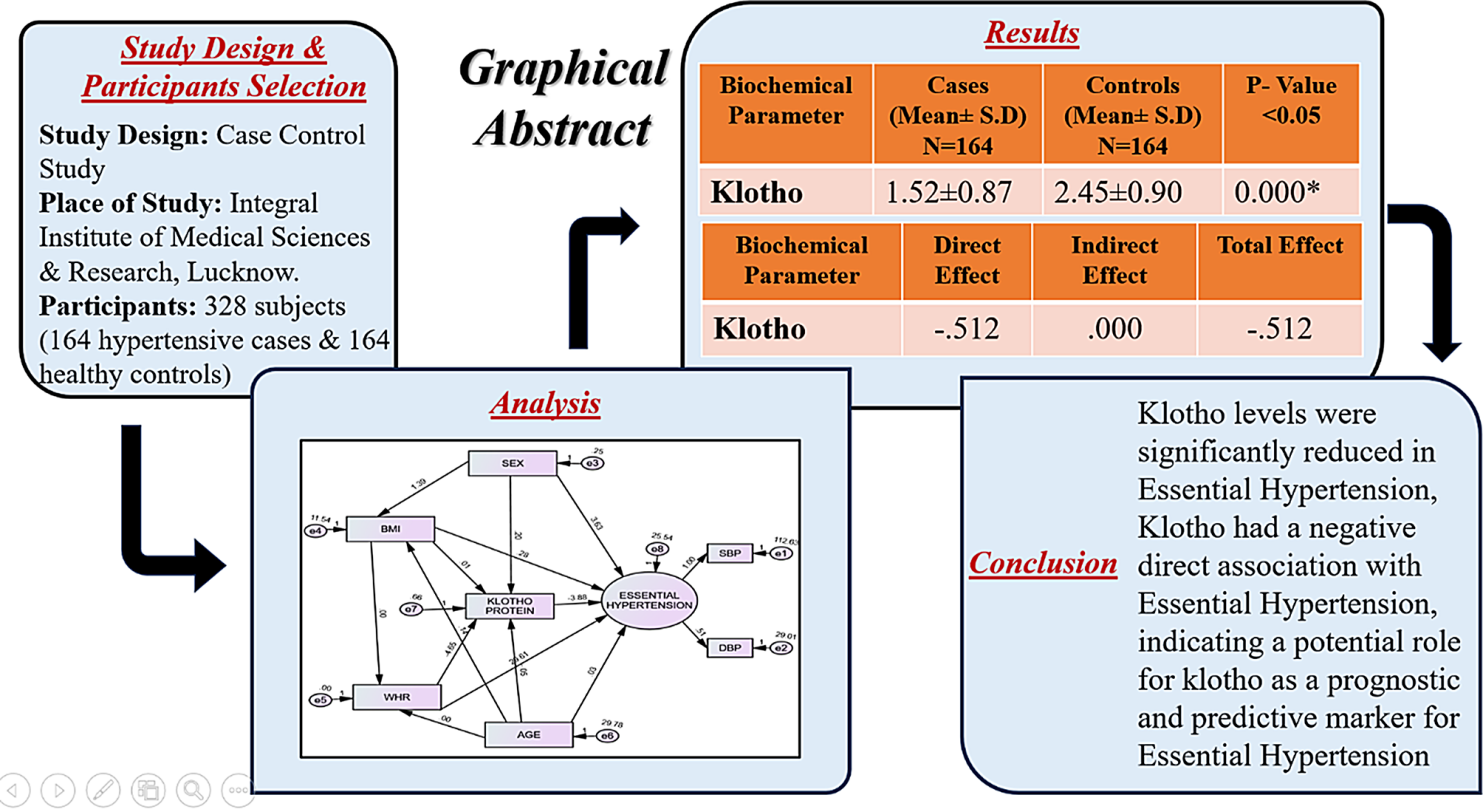

## Background

Essential hypertension is a prevalent cardiovascular condition with a multifactorial etiology, affecting 29% of the world’s adult population by 2025. It is a multifactorial disease resulting from genetic and environmental factors. Essential hypertension is a complex human disorder that is often studied but poorly understood [1]. Despite extensive research, the mechanisms underlying susceptibility and progression of essential hypertension are not fully understood. Recent research pointed out arterial aging as a substantial risk factor for the development of hypertension [2, 3].

Kuro-o [4] discovered an antiaging gene, klotho, which has been found to facilitate aging phenotypes and extend life expectancy. The klotho gene encode a single-pass transmembrane protein with intracellular, transmembrane, and extracellular domains, as well as a circulating α-klotho protein [5, 6]. α-Klotho protein is highly expressed within kidney tubules as a transmembrane protein that can be released as a soluble form into the circulation [4, 7]. This membrane-bound protein works with fibroblast growth factor-23, a bone-derived hormone that regulates urinary phosphate excretion [4, 6, 8, 9]. The circulating form of klotho (molecular mass of 130 kDa), detectable in plasma and urine, is named soluble or cleaved klotho. Klotho, a soluble protein, is formed through proteolytic cleavage of the extracellular part of the membrane-bound protein, consisting of two internal repeats, KL1 and KL2 [6, 10–13].

**Fig. 1:**
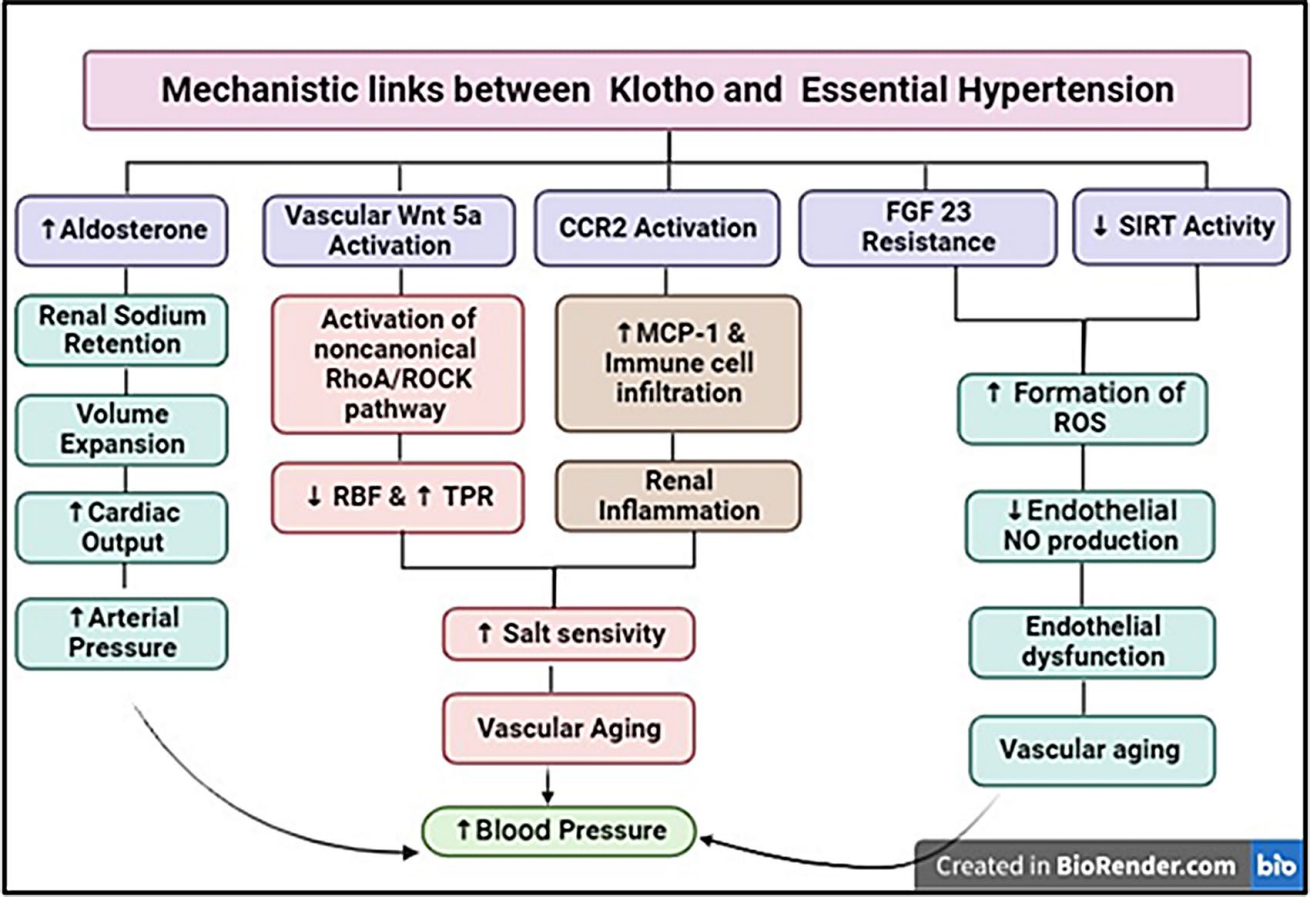
Machanism linking klotho deficiency with essential hypertension. Wnt, wingless-related integration site; CCR2, CC chemokine receptor 2; FGF23, fibroblast growth factor-23; SIRT, Sirtuin; ROCK, Rho-kinase; RBF, renal blood flow; TPR, total peripheral resistance; MCP-1, monocyte chemotactic protein-1; ROS, reactive oxygen species; NO, nitric oxide

α-Klotho, a circulating peptide hormone, influences various signaling pathways, including p53/p21, cyclic adenosine monophosphate, protein kinase C, wingless-related integration site, and insulin-like growth factor-1. Its antiaging effect is partly due to increased resistance to oxidative stress, which inhibits the insulin/insulin-like growth factor-1 signaling pathway, which is a key mechanism for essential hypertension development [6, 8]. In addition to oxidative stress, the other factors that contribute to the rise in blood pressure with aging include an increase in total peripheral resistance caused by salt intake, a decrease in the ability of the kidneys to excrete sodium, Vascular aging, endothelial dysfunction, and increased aldosterone levels (Fig. 1).

Klotho deficiency is associated with various molecular consequences that collectively promote arterial stiffening, vascular aging, and endothelial dysfunction, all leading to the development of hypertension. These consequences include the following: activation of vascular Rho-kinase via the noncanonical wingless-related integration site pathway; activation of CC chemokine receptor 2-mediated inflammatory processes contributing to salt-sensitive hypertension; upregulation of autophagy, which increases matrix metalloproteinase-9 and scleraxis activities, contributing to arterial stiffening and hypertension; vascular calcification and increased oxidative stress due to fibroblast growth factor-23 resistance [14–16]; and downregulation of sirtuin 1 activity, which decreases endothelial nitric oxide production and increases pro-oxidative, proinflammatory, and proapoptotic activities [1].

Although the studies mentioned above determined klotho deficiency as an independent factor for the development of hypertension in animal models and have suggested a proposed mechanism that links klotho protein deficiency with the risk of essential hypertension. Moreover, current knowledge of circulating serum klotho levels as a predictor for hypertension in humans is limited. Therefore, the present study was designed to examine the association of klotho protein with essential hypertension and also to assess its direct and indirect effect on essential hypertension.

## Methods

### Study participants

This case-control study was carried out in the Department of Biochemistry at Integral Institute of Medical Sciences and Research (Lucknow, India). A total of 328 subjects (164 hypertensive cases and 164 healthy controls) aged 35 to 60 years were enrolled in the study. Eligible candidates for the study were selected based on certain inclusion and exclusion criteria.

### Inclusion and exclusion criteria

Subjects were included as per Eighth Joint National Committee (JNC 8) criteria; blood pressure (˃140/90 mmHg) was considered hypertensive [17]. Subjects with blood pressure (< 120/80 mmHg) were considered healthy controls. Subjects with a history of liver disease, kidney disease, and cardiovascular diseases were excluded from the study. Subjects taking antihypertensive drugs and pregnant women were also excluded from the study. Demographic and clinical history details were recorded using a detailed structured patient proforma.

### Anthropometric measurements

Anthropometric measurements were recorded according to the World Health Organization’s STEPS (STEPwise approach to noncommunicable disease risk factor surveillance) guidelines. body mass index was calculated as body weight (kg) / height (m^2^).

### Blood pressure measurement

Subjects were asked to rest quietly for around 10 min before three subsequent measures were taken with a sphygmomanometer from each participant. Finally, the average of the two measurements was considered as the individual blood pressure value. Subjects having blood pressure more than (˃140/90 mmHg) were considered hypertensive.

### Sample collection and biochemical investigations

Under aseptic conditions, 2 mL blood was collected in the plain vial and was centrifuged at 3,000 rpm for 5 min to separate serum. The serum was further used for the estimation of calcium and klotho levels. Klotho protein activity was measured using the sandwich enzyme-linked immunosorbent assay method.

### Statistical analysis

The study used IBM SPSS Amos (IBM Corp) for data analysis, presenting mean ± standard deviation. An unpaired t-test was used to compare study parameters between cases and controls. A receiver operating characteristic (ROC) curve was created to examine the prognostic and predictive utility of serum klotho for essential hypertension. A structural equation model (SEM) was prepared to analyze the direct, indirect, and total effects of demographic, anthropometric, and biochemical profiles on essential hypertension. The cutoff criteria for the confirmatory factor analysis model fit included chi-square to df ratio < 3, goodness of fit index (GFI) > 0.90, adjusted GFI > 0.90, normed fit index (NFI) > 0.90, standardized root mean square residual (SRMR) < 0.08, and root mean square error of approximation (RMSEA) ≤ 0.05 [18, 19].

**Fig. 2:**
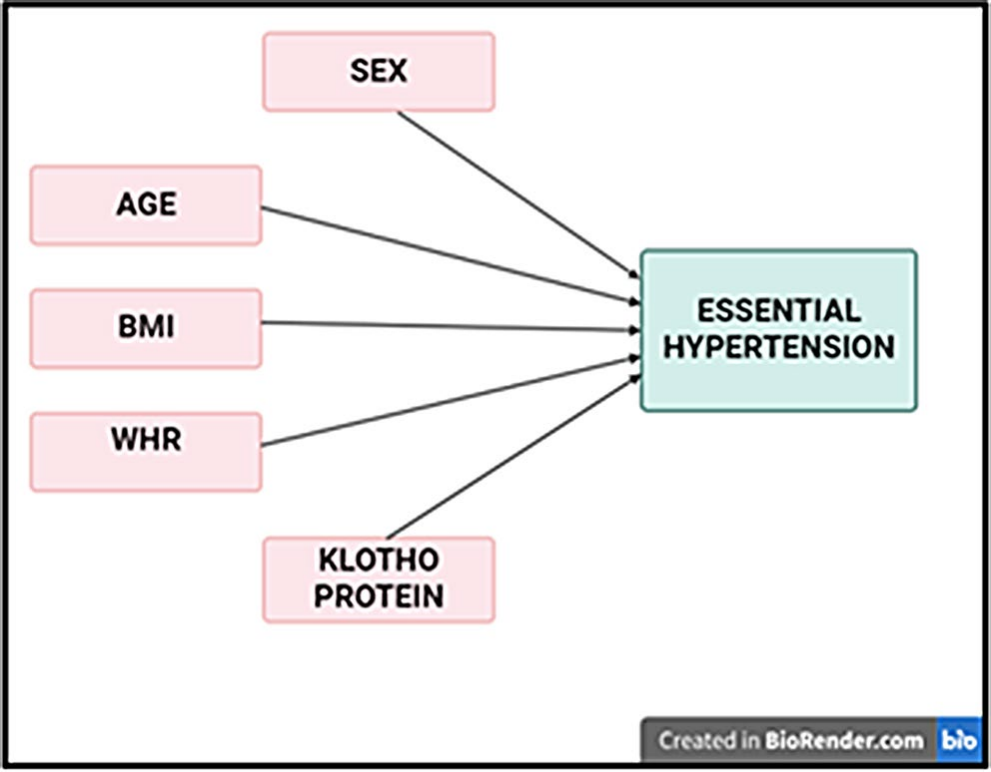
Conceptual model showing the association of demographic, anthropometric and biochemical profiles with essential hypertension

The conceptual model outlines the relationships between exogenous and endogenous variables and their association with essential hypertension. Exogenous variables include demographic profile (sex and age), anthropometric profile (body mass index and waist to hip ratio), and biochemical profile (klotho), while endogenous variables are systolic blood pressure and diastolic blood pressure. The model is translated into a statistical model using confirmatory factor analysis and initial SEM to test if the data fits the hypothesized measurement model (Fig. 2).

In the next step, SEM with maximum likelihood estimation was applied to assess the conceptual model. In SEM, the single-headed arrow represents a causal relationship or Regression, and the double-headed arrow represents correlation. The study used SEM to analyze the relationships between variables related to essential hypertension. The SRMR value measures the difference between the observed and model-implied covariance matrix, while the chi-square to df ratio gives chi-square estimates. The RMSEA measures the difference between the model and the observed covariance matrix, adjusting for model complexity. The P-close value assesses the likelihood of the observed data fitting the model, with P-values less than 0.05 indicating statistical significance.

## Results

### Participants characteristics

A total of 328 subjects were included in this case-control study. The results of the statistical analysis have been summarized in the tables. Demographic, anthropometric, and biochemical profiles of the subjects are presented in Table 1.

**Table 1:** Demographic, anthropometric, and biochemical profiles of the study groups (*n* = 328)

Variable	Essential hypertension group (*n* = 164)	Control group (*n* = 164)	*P*-value
Age (yr)	45.26 ± 5.47	43.78 ± 6.96	0.033^*^
Sex			>0.999
Male	81(49.4)	81(49.4)	
Female	83(50.6)	83(50.6)	
Body mass index (kg/m^2^)	24.38 ± 3.56	23.22 ± 3.47	0.003^*^
Waist to hip ratio	0.90 ± 0.03	0.91 ± 0.03	0.003^*^
Systolic blood pressure (mmHg)	154.54 ± 12.53	120.70 ± 9.41	<0.001
Diastolic blood pressure(mmHg)	93.39 ± 6.37	76.15 ± 7.18	<0.001
Klotho (ng/mL)	1.52 ± 0.87	2.45 ± 0.90	<0.001

Data are presented as mean ± standard deviation or number (%). ^*^P < 0.05

### Serum klotho and essential hypertension

**Fig. 3:**
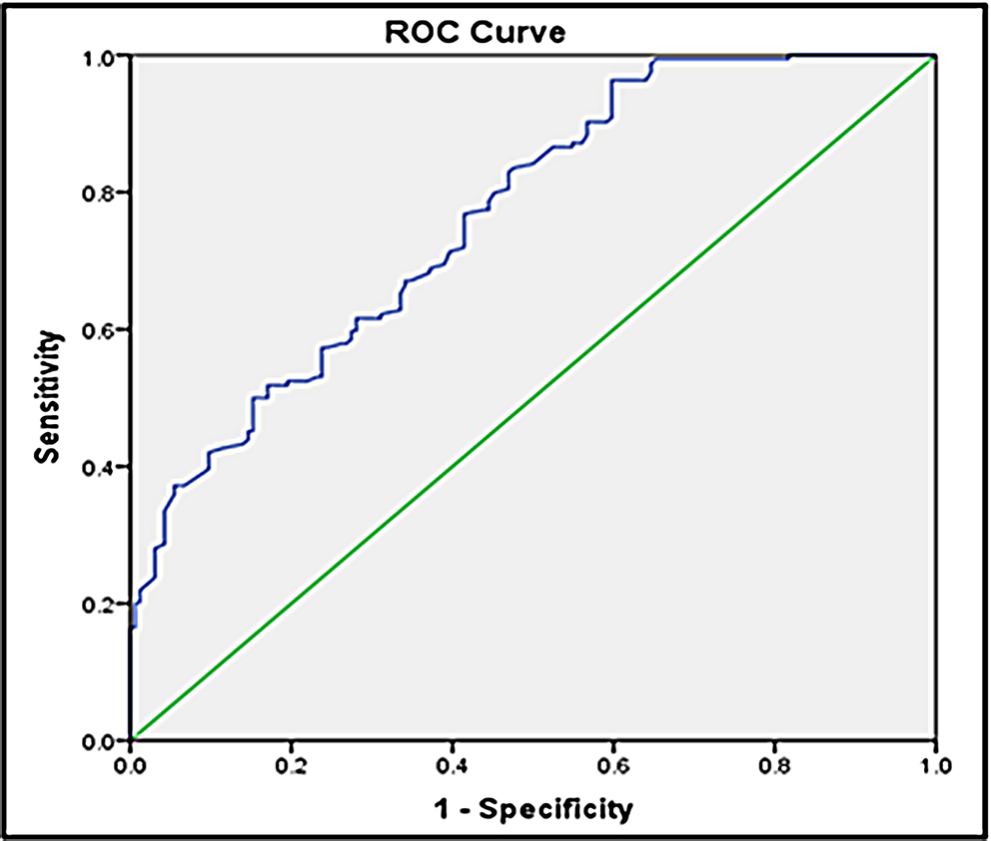
Receiver operating characteristic (ROC) curve for klotho protein predicting essential hypertension

According to statistical analysis, klotho levels were significantly reduced in patients with essential hypertension compared to the control (1.52 ± 0.87 vs. 2.45 ± 0.90, *P* < 0.001). The ROC curve of klotho with blood pressure is shown in Fig. 3. The area under the ROC curve for klotho and blood pressure was 0.765 (95% confidence interval, 0.716–0.815; *P* < 0.001).

## Results of the model structure

**Fig. 4:**
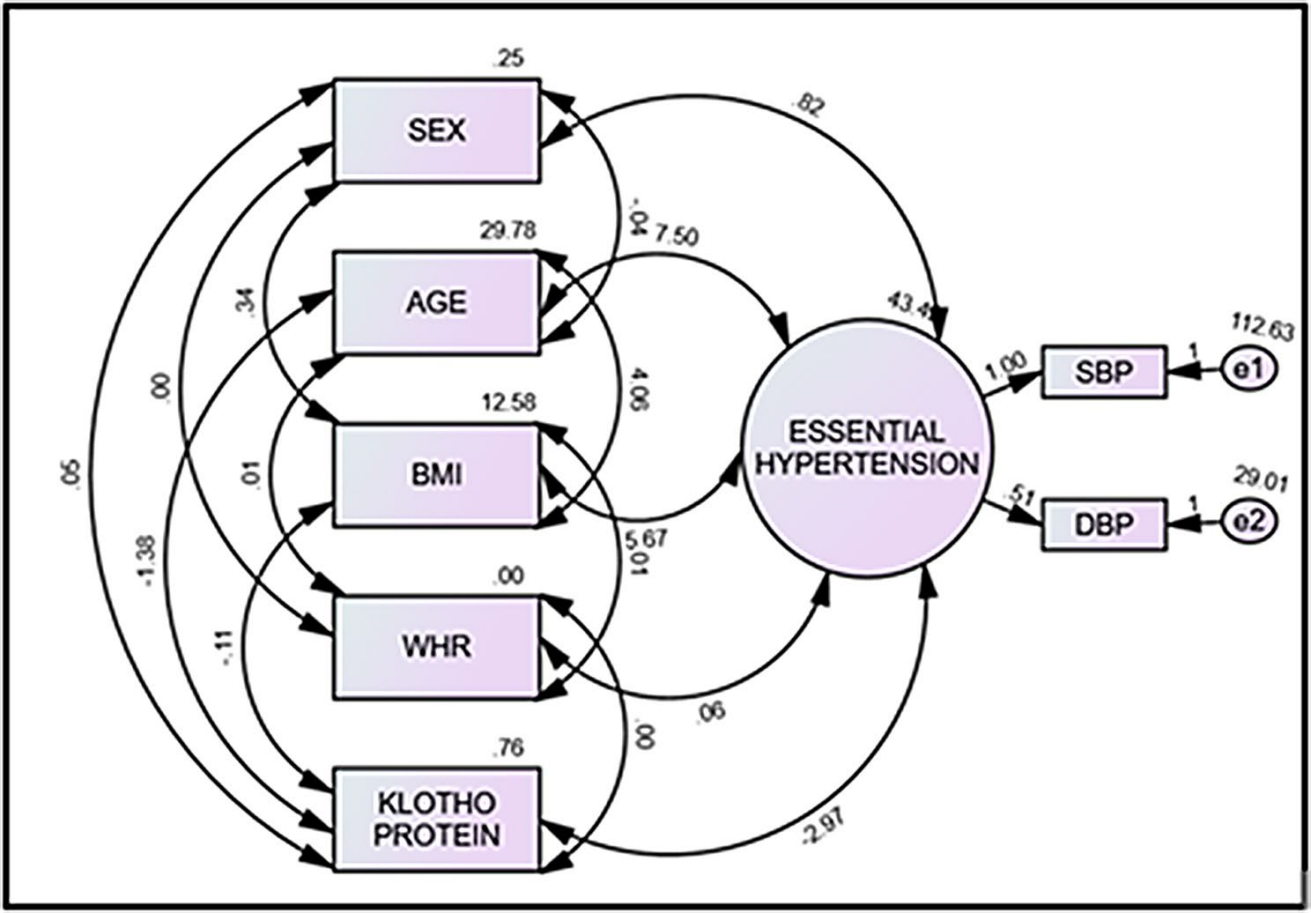
Structural equation model with an estimate of confirmatory factor analysis of demographic, anthropometric, and biochemical parameters with essential hypertension. BMI, body mass index; WHR, waist to hip ratio; SBP, systolic blood pressure; DBP, diastolic blood pressure

**Fig. 5:**
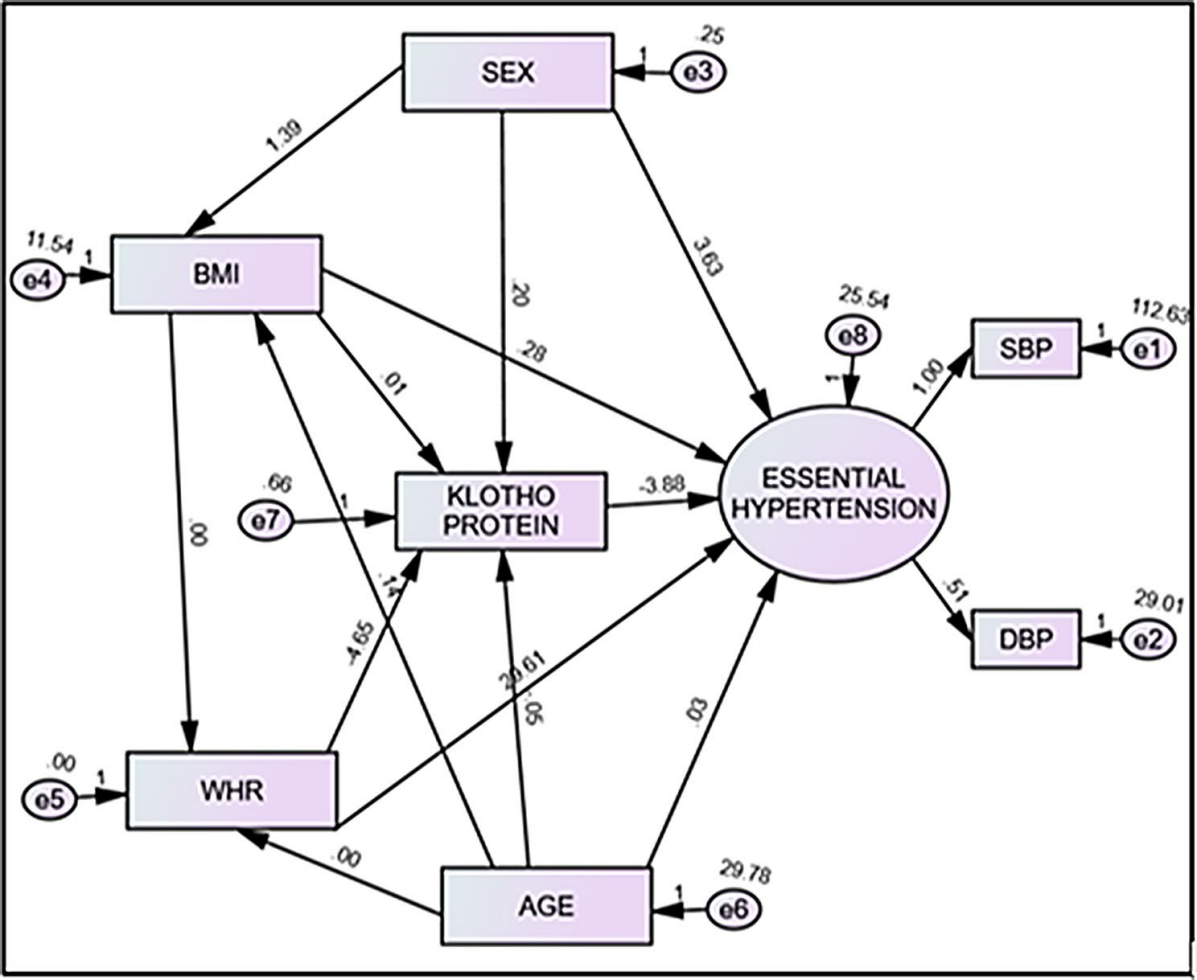
Structural equation model with estimates of direct, indirect, and total effect of independent variables on essential hypertension. BMI, body mass index; WHR, waist to hip ratio; SBP, systolic blood pressure; DBP, diastolic blood pressure

All indices in the model have suggested that the final model fitted the data reasonably shown in Table 2. The SEM showed a significant negative correlation between klotho and blood pressure in essential hypertension cases (Table 3; Fig. 4). The estimates of the model also showed the direct, indirect, and total effect of independent variables on desirable outcomes i.e., essential hypertension (Table 4; Fig. 5).

**Table 2:** Estimates of confirmatory factor analysis model fit

Measure	Estimate	Threshold
Chi-square to df ratio	1.153	<3
GFI	0.990	>0.90
Adjusted GFI	0.945	>0.90
Normed fit index	0.936	>0.90
SRMR	0.953	<0.08
RMSEA	0.031	≤0.05

GFI, goodness of fit index; SRMR, standardized root mean square residual; RMSEA, root mean square error of approximation

**Table 3:** Correlation between demographic, anthropometric, and biochemical profiles among hypertension cases

Variable	Estimate
Age and sex	− 0.014
Age and BMI	0.210
BMI and WHR	0.122
WHR and klotho	− 0.180
Sex and klotho	0.119
Age and klotho	− 0.291
BMI and klotho	− 0.036
Sex and WHR	0.037
Age and WHR	0.062
Sex and BMI	0.193
Essential hypertension and klotho	– 0.517
Essential hypertension and age	0.209
Essential hypertension and sex	0.248
Essential hypertension and WHR	0.265
Essential hypertension and BMI	0.243

BMI, body mass index; WHR, waist to hip ratio

**Table 4:** Direct, indirect, and total effects of independent variables on essential hypertension

Variable	Direct effect	Indirect effect	Total effect
Sex	0.275	− 0.025	0.251
Age (yr)	0.024	0.188	0.212
Body mass index (kg/m^2^)	0.149	0.015	0.163
Waist to hip ratio	0.143	0.087	0.229
Klotho	− 0.512	0	− 0.512

## Discussion

The major finding of the present study was klotho was significantly reduced in essential hypertension patients compared to controls. All indices in the SEM model have suggested that the final model fitted the data reasonably (chi-square to df ratio, 1.153; GFI, 0.990; adjusted GFI, 0.945; NFI, 0.936; SRMR, 0.953; RMSEA, 0.031). Also, a significant negative correlation of klotho with essential hypertension was observed. The area under the ROC curve value of 0.765 (95% confidence interval, 0.716–0.815; *P* < 0.001) for klotho and blood pressure showed the prognostic utility of klotho for essential hypertension.

Hypertension is one of the most challenging health problems in the world [20]. Several factors have been recognized as contributing to hypertension, including oxidative stress and aging. Soluble klotho is a circulating protein that acts as an antiaging protein. Soluble klotho has an important role in maintaining endothelial wall homeostasis and promoting the health of the vasculature [21–23]. In preclinical studies, klotho was found to be protective against the development of hypertension [24]. However, experimental studies have confirmed that soluble klotho may act as a humoral factor that protects the vascular system [15]. Also, experimental models have reported, that the absence of the klotho gene is linked to endothelial dysfunction and diffuse vascular calcification [22, 25].

Oxidative stress is a significant factor linked to hypertension, causing endothelial damage, vascular dysfunction, cardiovascular remodeling, renal dysfunction, and systemic inflammation [26]. Mishra et al. [27] in their study found that hypertensive patients are more likely to exhibit oxidative stress compared to controls. Endothelial dysfunction is an early stage in the onset and progression of cardiovascular disease caused by an imbalance in the release of vasodilator and vasoconstrictor substances. This disrupted equilibrium is predominantly due to the reduced bioavailability of nitric oxide because of its inactivation by reactive oxygen species [28]. Nitric oxide not only produces vasodilatation but also prevents atherogenic mechanisms by reducing smooth muscle cell proliferation and restricting the expression of adhesion molecules and platelet aggregation [29].

Blood pressure and klotho appear to be closely interrelated. Studies in animal models showed that α-klotho deficiency resulted in salt-sensitive hypertension [30]. Experimental evidence revealed that exogenous klotho administration in hypertensive rats’ results in decreased activation of the renin-angiotensin system and reduction in blood pressure [31], klotho deficiency in mice results in salt-sensitive hypertension [32], and klotho gene delivery attenuates hypertension and hypertensive kidney damage [31].

Human studies have shown a significant correlation between blood pressure and klotho protein levels, with higher klotho levels associated with lower risk of developing hypertension [25]. Early detection of low klotho concentrations and timely care can prevent mortality [33]. A cross-sectional study in postmenopausal women with hypertension found a significant inverse correlation between klotho concentration and hypertension, suggesting that serum klotho levels could be a useful biomarker for identifying women at risk of hypertension [34].

Despite these observations, the relationship of klotho with blood pressure has only been partially studied in humans [35, 36]. Studies have shown an inverse relationship between blood pressure salt sensitivity and levels of the protein klotho in essential hypertension patients. Furthermore, recent research suggests that there is a connection between klotho deficiency and the development of essential hypertension.

Thus, the study suggests more observational and clinical trial studies to be conducted in the future to corroborate the current study’s findings. The study also suggests that healthcare professionals should consider klotho as a predictive marker in aging-associated essential hypertension patients after certain clinical trials.

## Conclusions

Klotho levels were significantly reduced in essential hypertension cases compared to controls, Also, klotho had a negative direct association with essential hypertension indicating a potential role for klotho as a prognostic and predictive marker for essential hypertension. This suggests that klotho may have a role in the pathogenesis of essential hypertension. Understanding klotho’s role in essential hypertension may lead to the development of novel therapeutic strategies for this disease.

## Data Availability

Details of all data used for the study are mentioned in the Methods section of the manuscript.

## Abbreviations

GFI: Goodness of fit index
JNC 8: Eight Joint National Committee
NFI: Normed fit index
RMSEA: Root mean square error of approximation
ROC: Receiver operating characteristic
SEM: Structural equation model
SRMR: Standardized root mean square residual
STEPS: STEPwise approach to noncommunicable disease risk factor surveillance
